# Follicular thyroid carcinoma with an iliac wing metastasis – Rare case report at Bugando Medical Centre in Tanzania

**DOI:** 10.1016/j.ijscr.2021.106615

**Published:** 2021-11-22

**Authors:** Olivia Michael Kimario, Patric Ngoya, Osca Otman, Leonard Washington, Alicia Massenga, ZephaniaSaitabau Abraham

**Affiliations:** a Department of Otorhinolaryngology, Catholic University of Health and Allied Sciences, Mwanza, Tanzania; b Department of Radiology, Catholic University of Health and Allied Sciences, Mwanza, Tanzania; c Department of Pathology, Catholic University of Health and Allied Sciences, Mwanza, Tanzania; d Department of General Surgery, Catholic University of Health and Allied Sciences, Mwanza, Tanzania; e Department of Surgery, University of Dodoma, School of Medicine and Dentistry, Dodoma, Tanzania

**Keywords:** Follicular, Thyroid carcinoma, Metastasis, Iliac wing

## Abstract

Thyroid carcinoma is uncommon in our geographical setting. When it occurs the leading histological type is papillary thyroid carcinoma followed by follicular thyroid carcinoma which are differentiated thyroid carcinomas. Differentiated thyroid carcinomas usually have a good prognosis as compared to undifferentiated thyroid carcinomas. Follicular Thyroid Carcinoma usually presents with a solitary thyroid nodule with or without cervical lymphadenopathy. We present a 57 year old female with history of anterior neck swelling for 5 years and inability to walk using the left lower limb for 2 years. Total thyroidectomy and modified neck dissection was done. Histopathology results revealed follicular thyroid carcinoma. Patient was received radiochemotherapy treatment post-surgery. Follicular thyroid carcinoma may present with a symptomatic distant metastatic bony lesion as presented. It is important for clinicians to be aware this and carry out confirmatory relative investigations.

## Highlights

• Thyroid carcinoma is uncommon in our settings with the leading variant being papillary thyroid carcinoma.
• Differentiated thyroid carcinomas have a good prognosis compared to undifferentiated thyroid carcinomas.
• Metastatic involvement of iliac wing by follicular thyroid cancer remains to be a rare occurrence.
• Papillary thyroid cancer may metastasize to the lungs and bones.
• The pelvis is the second most common metastatic region for papillary thyroid cancer by the spine.

## 1 Introduction

Thyroid cancer is a relatively rare disease, accounting for approximately 1% of all malignant neoplasms, about 0.5% in men and 1.5% in women [1]. Various histological types of thyroid cancers have been identified, including differentiated thyroid cancers (DTC), hürthle-cell, undifferentiated carcinoma and medullary carcinoma. About 90% of thyroid cancers are differentiated, including both papillary (70–75%), and follicular (15–20%) cancers. Papillary DTC is characterized by indolence and localized spread, but may metastasize to the lungs and bones. Follicular DTC is known to preferentially metastasize to the lungs and bones via hematogenous spread [2]. Bone metastases have been reported in 2.3–12.7% of patients with DTC [3]. Most bone metastases occur in areas of high blood flow, including the red marrow regions of the axial skeleton, including the vertebrae (42–52%), femur (9–20%), skull (2–16%) and pelvis (5–13%) [4].

The pelvis is the second most common region of the metastatic bone lesion following the spine. Metastatic tumors of the pelvis may cause pain and a significant loss of function and weight-bearing capacity. Due to the relatively large size of the pelvic cavity, the elastic nature of the organs it contains, and its surrounding muscles, tumors at that site usually reach considerable size before causing symptoms. While some locations of metastases within the pelvis have no impact on pelvic stability and function (e.g., ilium, pubis), lesions of the posterior ilium may pose a threat to lumbosacral integrity while lesions involving the acetabulum may profoundly impair hip function and the weight-bearing capacity of the lower extremity [5].

DTC is one of the most curable cancers [6]. DTCs are characterized by a slowly progressive course, and have a 10-year survival rate of 80–95% [7]. However, the occurrence of distant metastases reduces the overall 10-year survival rate to 40% [8]. Previous studies have reported that 25% of metastases were to the bone, 49% to the lung and 15% to both. Bone metastases have been reported in 2–13% of patients with DTC, being significantly more frequent in patients with follicular cancer (7–28%) than in those with papillary cancer (1.4–7%) [9], [10]. Patients with thyroid cancer and bone metastases have a poor prognosis, with 10-year survival rates ranging from 0 to 34% [11]. Complete resection of bone metastases of DTC has been associated with a significant improvement in survival [3], [12].

## 2 Case presentation

57 year old female referred from Kitete Regional Referral Hospital, Tabora with main complains of anterior neck swelling for 5 years and inability to walk using the left lower limb for 2 years.

The anterior neck swelling increased gradually in size with time associated with pressure symptoms such as difficulty in swallowing however no change in voice. Patient provided history of cooking using firewood however, no history of radiation exposure nor having a close relative with goiter.

History of inability to walk using the left lower limb also started gradually by having limited movement and later inability to bear weight on the limb, when done so results to pain. No history of trauma reported by patient.

**Fig. 1:**
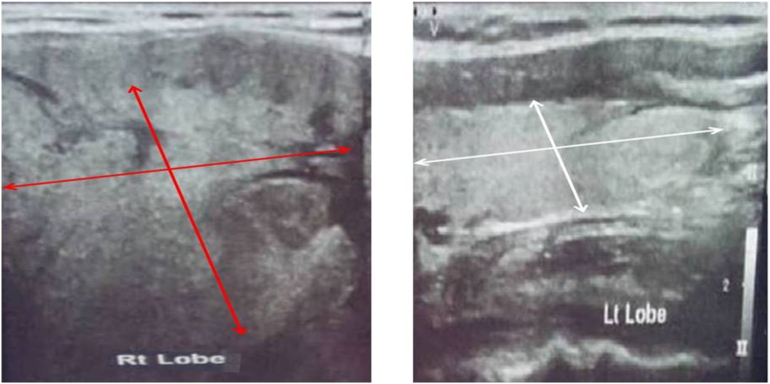
Thyroid ultrasound illustrated an enlarged right thyroid lobe (red arrows) with a heterogeneous nodular parenchymal echo relative to the left thyroid lobe (white arrows) which had a normal size and homogenous parenchymal echo.

On general examination, patient was oriented to time, place and people, not wasted, not jaundiced, not cyanotic, not pale, no enlarged cervical lymph nodes, no lower limb edema. Vital signs were normal (Fig. 1).

**Fig. 2:**
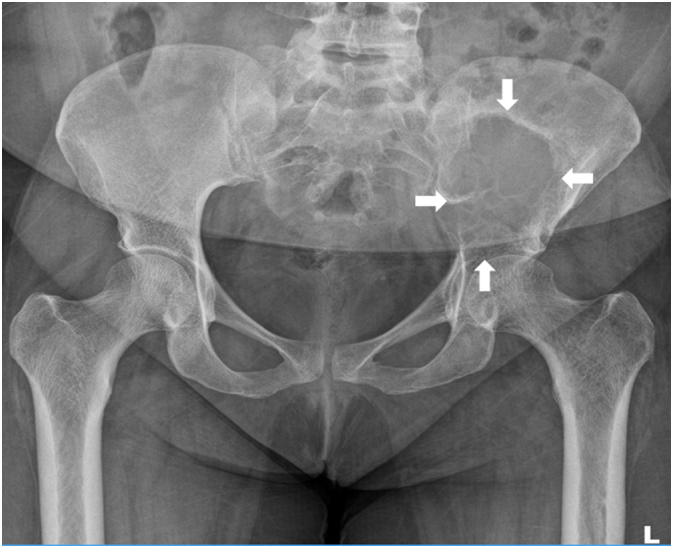
X-ray of the pelvis demonstrated a large lytic bubbly lesion (solid white arrows) within the left iliac wing extending to involve the left acetabulum but sparing the left femur demonstrating a lobulated sclerotic superior border and ill defined inferior, medial and lateral borders suggestive of a bony metastasis in the context of a malignant thyroid mass.

On local examination, patient had a right sided anterior neck mass which moved up on swallowing, it was mobile, firm, non tender and measured about 4 cm by 5 cm in size. The mass involved only the right thyroid lobe - the left thyroid lobe was not palpable. There was neither shift of trachea nor retro-sternal extension. There was no vocal cord palsy on fiber-optic laryngoscopy (Fig. 2).

**Fig. 3:**
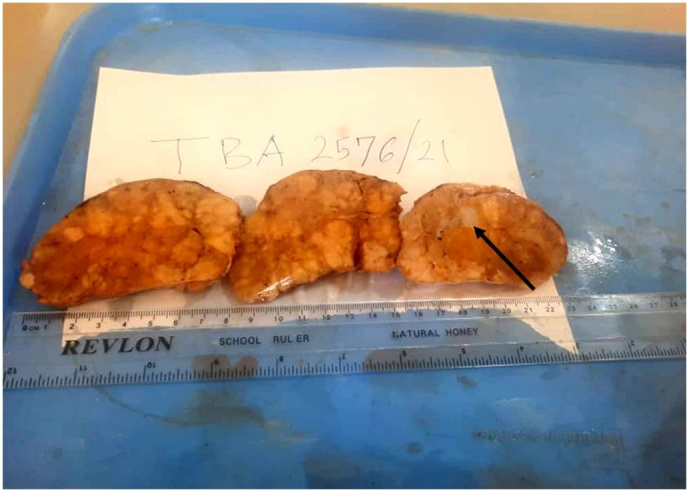
Shows a gross thyroidectomy specimen (black solid arrow) with multiple nodules of variable sizes infiltrating the capsule.

**Fig. 4:**
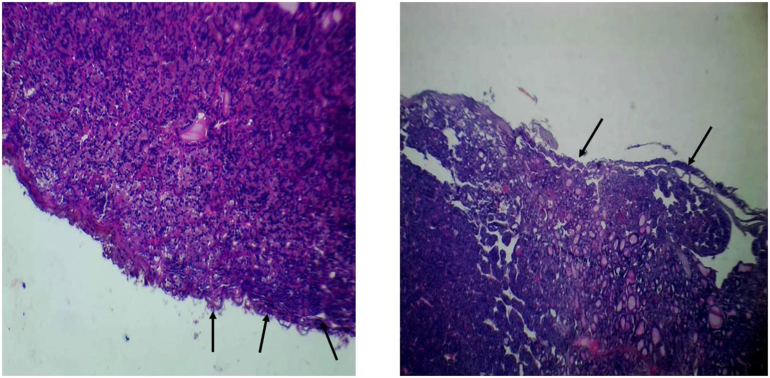
Shows hematoxylin and eosin sections (×4 hpf) of numerous microfollicles forming diffuse solid tumor infiltrates into the capsule (black solid arrows).

**Fig. 5:**
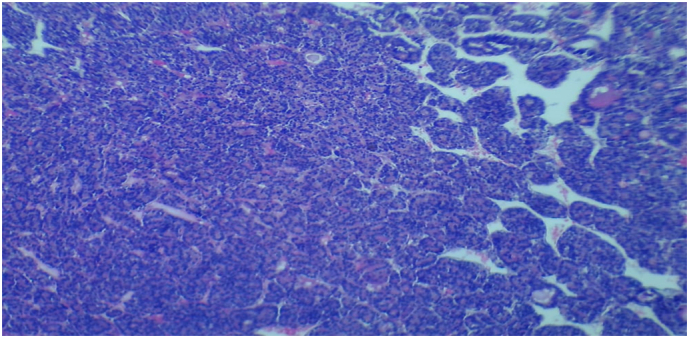
Shows a hematoxylin and eosin section (×10 hpf) of the solid microfollicles some with scanty colloid material formed a trabecular arrangement.

**Fig. 6:**
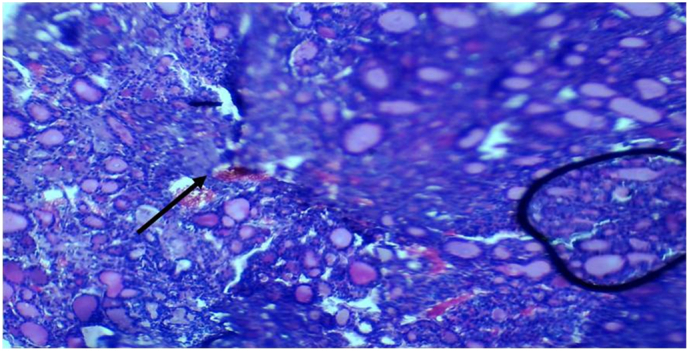
Shows a hematoxylin and eosin section (×40 hpf) of the vascular invasion (black solid arrow).

We had a preoperative diagnosis of thyroid carcinoma whereby total thyroidectomy and modified neck dissection was performed and the specimen taken for histopathological analysis. The histopathology results (Fig. 3, Fig. 4, Fig. 5, Fig. 6) had a confirmatory diagnosis of follicular thyroid carcinoma.

## 3 Discussion

Thyroid carcinoma is uncommon in our Tanzania although when it does occur major histological type is papillary thyroid carcinoma followed by the follicular thyroid carcinoma. Both of are well differentiated tumors with a good prognosis upon treatment. Females are more commonly affected as shown in our case presentation [1].

The patient had a confirmatory histological diagnosis of follicular thyroid carcinoma with a metastatic lesion to the left iliac wing involving the left acetabulum which rich in blood as has been described [4].

The pelvis is the second most common region of the metastatic bone involvement followed by the spine. Metastases in the pelvis may cause pain and a significant loss of function and weight-bearing capacity. Due to the relatively large size of the pelvic cavity, the elastic nature of the organs it contains, and its surrounding muscles, tumors at that site usually reach considerable size before causing symptoms. While some locations of metastases within the pelvis have no impact on pelvic stability and function (e.g., ilium, pubis), tumors of the posterior ilium may pose a threat to the lumbar-sacral integrity, and tumors of the acetabulum may profoundly impair hip function and the weight-bearing capacity of the lower extremity [5] as presented in our case.

Despite intra-operative hemorrhage, the patient was managed successfully by total thyroidectomy and modified neck dissection and later underwent radiochemotherapy treatment at the Oncology Department. Patient continued follow up at the out-patient clinic after treatment.

## 4 Conclusion

Follicular thyroid carcinoma may present with a symptomatic distant metastatic bony lesion as presented.

## Recommendation

It is important for clinicians to be aware this and carry out confirmatory relative investigations.

## Ethical approval

Ethical approval was obtained from Institutional Ethics and Research Committee.

## Funding

None.

## Guarantor

Dr. Olivia Michael Kimario takes full responsibility of the work.

## Research registration number

CREC/487b/2021.

## CRediT authorship contribution statement

OMK-Conceptualization, methodology, writing original draft
PN-Conceptualization and reviewing the prepared original draft of the manuscript
OO-Conceptualization, methodology and reviewing the prepared original draft of the manuscript
LW-Conceptualization, methodology and reviewing the prepared original draft of the manuscript
AM-Conceptualization, methodology and reviewing the prepared original draft of the manuscript
ZSA-Conceptualization, methodology and reviewing the prepared original draft of the manuscript.

## Declaration of competing interest

None.
